# Targeting Neutrophils in Sepsis: From Mechanism to Translation

**DOI:** 10.3389/fphar.2021.644270

**Published:** 2021-04-12

**Authors:** Xiaofei Shen, Ke Cao, Yang Zhao, Junfeng Du

**Affiliations:** ^1^ Faculty of Hepato-Biliary-Pancreatic Surgery, Chinese People’s Liberation Army (PLA) General Hospital, Beijing, China; ^2^ Department of Critical Care Medicine, Nanjing Drum Tower Hospital, The Affiliated Hospital of Nanjing University Medical School, Nanjing, China; ^3^ State Key Laboratory of Membrane Biology, Institute of Zoology, Chinese Academy of Sciences, Beijing, China; ^4^ Medical Department of General Surgery, The 1st Medical Center of Chinese PLA General Hospital, Beijing, China; ^5^ Department of General Surgery, The 7th Medical Center, Chinese PLA General Hospital, Beijing, China; ^6^ The Second School of Clinical Medicine, Southern Medical University, Guangdong, China

**Keywords:** neutrophil, sepsis, therapeutic target, Signaling pathway, translational medicine

## Abstract

Sepsis is a life-threatening condition caused by a dysregulated host response to infection. Although our understanding in the pathophysiological features of sepsis has increased significantly during the past decades, there is still lack of specific treatment for sepsis. Neutrophils are important regulators against invading pathogens, and their role during sepsis has been studied extensively. It has been suggested that the migration, the antimicrobial activity, and the function of neutrophil extracellular traps (NETs) have all been impaired during sepsis, which results in an inappropriate response to primary infection and potentially increase the susceptibility to secondary infection. On the other hand, accumulating evidence has shown that the reversal or restoration of neutrophil function can promote bacterial clearance and improve sepsis outcome, supporting the idea that targeting neutrophils may be a promising strategy for sepsis treatment. In this review, we will give an overview of the role of neutrophils during sepsis and discuss the potential therapeutic strategy targeting neutrophils.

## Introduction

Sepsis is a highly complex disease caused by the dysregulated response to infection, and it is also one of the common causes of death in patients with acute and critical illness. With the extensive development of the Surviving Sepsis Campaign guidelines in clinical diagnosis and treatment (Rhodes et al., 2017), the use of antibiotics, early fluid resuscitation, and the standardized application of multiple organ function support methods within 1 h have downregulated the short-term fatality rate to about 20% in patients with sepsis (Fleischmann et al., 2016). With an increased understanding in the pathophysiological process of sepsis, sepsis has now been redefined as life-threatening organ failure caused by host immune response imbalance after infection (Singer et al., 2016). Based on the fundamental characteristics of sepsis, how to modulate immune response to infection during the whole process of sepsis and to maintain immune homeostasis have always been the central problems for sepsis treatment. Although no specific treatment for sepsis has been developed, accumulating evidence has suggested a beneficial role of immune modulation therapy in sepsis treatment (Hotchkiss et al., 2019; Rubio et al., 2019).

Neutrophils, one of the most abundant immune subsets in the peripheral, represent the first line of defense against invading pathogens. Microbial infection leads to the generation of granulocytes in the bone marrow and subsequently a release of both immature and mature forms of neutrophils into the peripheral blood (Manz and Boettcher, 2014). Excessive immature form of neutrophils in the peripheral is a hallmark of systemic inflammatory response syndrome, and is also related to clinical deterioration in patients with sepsis (Daix et al., 2018). Impaired migration to the infection site and dysregulated function capacity, as well as a prolonged presence of neutrophil extracellular traps in vasculature or tissues (Camicia et al., 2014; Shen et al., 2017a), have been identified during the process of sepsis, which is also critical for sepsis progression. Therefore, a better understanding in the impact of immune responses between different forms of neutrophils and the molecular control of endothelial and tissue damage mediated by neutrophils may provide beneficial prospects for future development of immune modulatory therapies targeting neutrophils.

## Role of Neutrophils in Sepsis

### Dysregulated Migration of Neutrophils

The migration of neutrophils to infection sites is critical for their antimicrobial function. It has been suggested that all the phases responsible for neutrophil migration have been impaired during sepsis, including mobilization and release from the bone marrow, migration and rolling, adherence, and transmigration (Shen et al., 2017a). The release of neutrophils into peripheral blood is tightly controlled by C-X-C chemokine receptor (CXCR)4 interacting with (CXCL)12 (Shen et al., 2017a). During sepsis, downregulation of CXCL12 occurs, which leads to an increased release of neutrophils into peripheral blood (Delano et al., 2011a). After being released into peripheral blood, the deformability capacity ensures effective rolling of neutrophils, and integrins expressed on neutrophils such as LFA-1 and Mac-1 display low-affinity adhesion with selections expressed on vascular endothelium, which promotes the margination of neutrophils (Lerman and Kim, 2015). Bacterial products upregulate the expression of β1 and/or β2 integrins on neutrophils during sepsis, as well as increased expression profiles of adhesion molecules including ICAM-1 and VCAM-1, leading to a high-affinity adhesion of neutrophils with vascular endothelium (Kovach and Standiford, 2012; Lerman and Kim, 2015). Stimulation by pro-inflammatory mediators such as fMLP or TNF-α also drives the accumulation of F-actin below the cell membrane in a peroxisome proliferator-activated receptor gamma (PPARγ)-dependent manner, leading to reduced deformability (Reddy et al., 2008). As a result, reduced deformability and increased adhesion with vascular endothelium promote the retention of neutrophils in the vascular compartment, resulting in vascular occlusion and tissue ischemia (Saito et al., 2002).

The transmigration of neutrophils from the vascular compartment into the infection site is driven by the interaction between chemoattractants and CXCR2 on neutrophils, which is downregulated during sepsis progression. Mechanism studies have shown that prolonged or repeated stimulation from chemoattractants leads to the internalization of CXCR2 mediated by the activation of G-protein coupled receptors (GPCRs) in a β-arrestin– and calthrin-dependent manner (Lefkowitz and Shenoy, 2005; Lee et al., 2015). Negative regulators that can prevent the internalization of CXCR2 have also been suggested by our group and others, which may provide potential targets for future drug development (Martin et al., 2010; Shen et al., 2017b; Shen et al., 2017c). In addition to the impaired transmigration into infection sites, inappropriate migration of neutrophils to remote tissue and/or organ is also a major clinical feature during sepsis, which has been suggested to be mediated by CCR2 (Souto et al., 2011). Recently, the ability of neutrophils to retro-transmigrate into the bloodstream has been identified in mice (Wang et al., 2017). Infiltrated neutrophils interact with endothelial cells *via* CD11b and release neutrophil elastase, which degrades the JAM-C (Colom et al., 2015), allowing their circulation back to the bloodstream. The upregulation of CXCR4 on neutrophils in the bloodstream drives them to migrate to the lung (Wang et al., 2017), which may represent another potential mechanism for the induction of remote tissue injury mediated by neutrophils during sepsis. Moreover, retro-transmigrated neutrophils express ICAM-1 and display effective bacterial phagocytosis (Silvestre-Roig et al., 2016). However, these neutrophils are also associated with secondary organ injury at the same time (Woodfin et al., 2016).

## Impaired Antimicrobial Activity of Neutrophils in Sepsis

### Recognition of Pathogen or Pathogen Products

Once neutrophils have found and recognized an invading pathogen, they exert their antimicrobial function through phagocytosis, and subsequently pathogen killing, which occurs in the phagolysosome (Leliefeld et al., 2016). Two antimicrobial mechanisms have been suggested, including the oxidative killing and granule product–mediated killing. The recognition of pathogen or pathogen products by neutrophils also has an impact on the following antimicrobial activities, among which Toll-like receptors (TLRs) are suggested to play a critical role during these processes. Toll-like receptors signal the downstream release of antimicrobial peptides, pro-inflammatory cytokines, and chemokines, and also promote the generation of reactive oxygen species (ROS) through NF-κB and mitogen-activated protein kinase (MAPK) pathways (Kovach and Standiford, 2012). However, prolonged or repeated activation of TLRs also leads to the tolerance of the TLR signaling pathway, partly mediated by the downregulation of TLRs expressed on neutrophils. In addition, inhibitors of TLR signaling such as IRAK-M are upregulated during sepsis progression (Xiong and Medvedev, 2011), and the expression of NF-κB inhibitory molecule NFκBIA is also increased in human septic neutrophils (Tang et al., 2007). Therefore, the septic milieu alters the TLR signaling and promotes the hyporesponsiveness of neutrophils, which may trigger the inhibition of immune response against secondary infection.

### Antimicrobial Function

Both the oxidant activity and phagocytosis capacity of neutrophils have been suggested to be impaired during sepsis (Delano et al., 2011b; Mishra et al., 2012; Demaret et al., 2015). Gene clusters related to immune modulation genes, inflammatory genes, and genes required for ROS production have been identified to be suppressed in patients with sepsis (Tang et al., 2007). Kinases activity, which is responsive for the signaling cascades of neutrophil function, has also been suggested to be widely impaired in patients with sepsis (Hoogendijk et al., 2019). As mentioned above, neutrophils kill invading pathogens through digestive proteases in the phagolysosome, which is regulated by intra-phagosomal pH (Levine et al., 2015). During inflammation, ROS production in neutrophils leads to acidification of the phagosome, which results in impaired killing of ingested microbes (Kovach and Standiford, 2012). Consistent with this, acidification of peritoneal neutrophils was associated with mortality in the mouse sepsis model (Chiswick et al., 2015). In addition, serine proteases released by neutrophils can cleave complement receptors such as C5aR on neutrophils, which lead to a defect in neutrophil phagocytosis partly due to their comprised capacity of finding opsonized targets (Morris et al., 2011). Despite intrinsic regulation of antimicrobial function in neutrophils, environmental stimulus can also influence the antimicrobial capacity of neutrophils during sepsis. Mediators expressed by bacteria and host-derived factors have shown to impair neutrophil phagocytosis through limiting complement-mediated opsonization (Mishra et al., 2012). Cytokines such as resistin can also inhibit neutrophil killing capacity through reducing F-actin polymerization and suppression of the oxidative burst (Bonavia et al., 2017; Miller et al., 2019).

### Detrimental Effects of Improper Activation of Neutrophils

In addition to the impaired antimicrobial activities, it is likely that both killing mechanisms shift during the course of acute inflammation owing to the dysregulated activation of neutrophils. The proper generation of ROS is critical for the oxygen-dependent antimicrobial mechanism. In patients with fatal sepsis, markedly increased production of ROS in neutrophils was observed (Santos et al., 2012). Uncontrolled release of ROS accumulating in vascular beds can contribute to the loss of endothelial barrier integrity and subsequent vascular leakage, leading to organ injury such as acute lung injury (Fox et al., 2013; Zhao et al., 2020). In line with these findings, patients with increased ROS production were more prone to develop ARDS than control patients (Kellner et al., 2017). Granule products are mainly responsible for the non-oxidative killing mechanisms of neutrophils. However, during sepsis process, surface receptors responsible for neutrophil extravasation, homing, and activation mediate uncontrolled activation of neutrophils (Leliefeld et al., 2016). During this process, granules fuse with the plasma membrane, releasing their content into the environment. Excessive extracellular degranulation and the release of neutrophil proteases primarily reserved in granule resulted in collateral tissue damage (Martin-Fernandez et al., 2020). More tissue damage also led to increased influx and inappropriate activation of neutrophils, which together resulted in continuous tissue destruction (Laforge et al., 2020).

## Life Span and Immature Form of Neutrophils

### Increased Life Span of Neutrophils

The life span of circulating human neutrophils is estimated to be 5.4 days (Pillay et al., 2010), which provides opportunities for neutrophils to undergo phenotypic and functional changes. After infiltrating into tissues during acute inflammation such as sepsis, tissue-derived signals including cytokines and environmental factors (Silvestre-Roig et al., 2016) reduce neutrophil apoptosis and increase their life span to an extent that is currently unknown. Life span extension during inflammatory conditions also increases the chance for neutrophils to undergo phenotypic and functional changes. It has been suggested that the low-oxygen environment in tissue during inflammation drives hypoxia-inducible factor-dependent activation of neutrophil pro-survival pathways and has a direct impact on the bactericidal activity of neutrophils (Thompson et al., 2014). Endotoxin could also increase the levels of various gene transcripts in neutrophils, which results in the suppression of neutrophil apoptosis (de Kleijn et al., 2012). Because both mature and immature forms (discussed in the following section) of neutrophils are present during sepsis, detailed analyses of the apoptosis level of different forms of neutrophils may help to better understand the biological characteristics of neutrophils during sepsis. It is noteworthy that delayed apoptosis of neutrophils but accelerated apoptosis of other adaptive immune cells may impair the homeostasis of immunity, and increased life span also offers the chance for neutrophils to exert other immunoregulatory functions such as inducing T-cell apoptosis. Taken together, all these results may be a new reason for immune paralysis found in sepsis. The therapeutic strategy targeting delayed apoptosis of neutrophils has been tried recently, which has provided promising results (Zhang et al., 2019).

### Generation of Immature Form of Neutrophils

As the most abundant immune cells in the peripheral with a relatively short half-life, neutrophils undergo constant replenishment from the bone marrow to peripheral blood (Lahoz-Beneytez et al., 2016). The developmental path and functional properties of neutrophils in the bone marrow include granulocyte–monocyte progenitor (GMP) differentiating into neutrophil precursor population (Silvestre-Roig et al., 2016), which can further give rise to an intermediate immature population and subsequently the mature population (Evrard et al., 2018). In a mice sepsis model, increased neutrophil precursors were identified both in the bone marrow and spleen (Evrard et al., 2018), and the local environment further potentiates the expanded pool of neutrophils. Increased amounts of granulocyte colony-stimulating factor (G-CSF) can mobilize neutrophils from the bone marrow into the blood and upregulate the expression of chemokines such as CXCL1 (Kohler et al., 2011). As a result, both mature and immature neutrophils were driven to peripheral blood, which has been confirmed in patients with sepsis, although detailed mechanism for the presence of immature neutrophils is still lacking (Drifte et al., 2013; Hampson et al., 2017). CXCR2 is a distinguishable marker for circulating mature neutrophils as mature neutrophils downregulate the expression of CXCR4 and upregulate the expression of CXCR2 during the process of mobilization from the bone marrow to peripheral blood. Currently, it is unclear how to interpret the presence of immature cells in the bloodstream, which might be a compensatory response initiated by the depletion of mature neutrophils in the bone marrow or a dedicated inflammatory reaction to bacterial stimulus. Since these immature neutrophils also display a pronounced decrease of various receptors when compared to their mature counterparts, the first hypothesis is more likely to occur.

As previously described, a compromised expression of CXCR2 was identified in neutrophils of septic patients. However, since immature neutrophils also displayed low or negative expression of CXCR2 (Evrard et al., 2018), the downregulation of CXCR2 on neutrophils in patients with sepsis may also partly attribute to the presence of immature neutrophils. Therefore, future analysis on neutrophil migration and function should further take the variations in neutrophil forms into account. Interestingly, both mature and immature neutrophils were shown to display identical capacity of migration into tissues, at least in a sterile inflammation model (Evrard et al., 2018). In a human model of experimental endotoxemia, immature neutrophils also exhibited efficient migration (van Grinsven et al., 2019). With regard to antimicrobial function, immature neutrophils were shown to have decreased phagocytic capacity (Taneja et al., 2008) and reduced antimicrobial function (Danikas et al., 2008). These results were consistent with the recent finding using single-cell transcriptome profiling, which suggested that the dynamics of the oxidase complex subunits varied through neutrophil differentiation, with minimum activation-triggered NADPH oxidase activation in immature neutrophils (Xie et al., 2020). Thus, more detailed studies on the function of distinct subpopulations of neutrophils are needed, which may help to better understand their diversity and critical roles in severe inflammation such as sepsis.

### Suppressive Function of Neutrophils in Sepsis

Although neutrophils have long been recognized as effector cells for the eradication of bacteria and fungi, accumulating results have suggested their immune modulatory function in sepsis. Serine proteases released by neutrophils cleave CD14 on monocytes, which is necessary for recognition of lipopolysaccharide by TLR4 (Le-Barillec et al., 1999). Neutrophil elastase also reduces the expression of co-stimulatory molecules on dendritic cells (Roghanian et al., 2006), which limits a proper Th1 response. In acute systemic inflammation induced by endotoxin challenge, human mature neutrophils mediated the suppression of T-cell proliferation in an integrin Mac-1– and ROS-dependent manner (Pillay et al., 2012). Similar neutrophils were also found in septic shock patients, which can suppress T-cell function through the expression of arginase-1 (Darcy et al., 2014). Furthermore, neutrophils isolated from septic patients can upregulate the expression of PD-L1 in an interferon-gamma (IFN-γ)-dependent process, which induced the apoptosis of T cells (de Kleijn et al., 2013; Langereis et al., 2017). Taken together, these results suggested that the function of neutrophils should be tightly controlled to enhance their antibacterial function and at the same time also to prevent their immunosuppressive function on adaptive immunity.

### Role of Neutrophil Extracellular Traps in Sepsis

Neutrophils can exert their function through the formation of neutrophil extracellular traps (NETs). NETs are composed of a network of chromatin fibers containing granules of antimicrobial peptides and enzymes including myeloperoxidase, elastase, and cathepsin G (Brinkmann et al., 2004). Due to their structure features, it is speculated that the function of NETs is to capture and kill pathogens extracellularly, and also to prevent bacterial dissemination. Evidence from human and animal models further confirmed this, and it is now believed that NET is a critical antimicrobial mechanism used by neutrophils despite phagolysosomes and degranulation (Papayannopoulos, 2018). The formation of NETs was first described by Brinkmann et al. (2004), who showed that neutrophils stimulated with PMA, IL-8, or LPS could release NETs. Following studies have further revealed a wide range of stimuli that are capable of inducing the formation of NETs. After challenging with different stimuli, two forms of NETosis were introduced, including suicidal NETosis and vital NETosis (Yipp and Kubes, 2013). In suicidal NETosis, NAPDH-dependent ROS production leads to peptidyl arginine deaminase 4 (PAD4) activation, which results in chromatin decondensation and the following release of cell-free DNA through membrane pores and cellular lysis (Wang et al., 2009; Leshner et al., 2012). In vital NETosis, neutrophils challenged with bacterial form budding DNA-containing vesicles from the nuclear envelop, which fuse with the plasma membrane to release DNA to the extracellular space without a loss in the integrity of the nuclear or plasma membrane (McDonald et al., 2012; Yipp et al., 2012). Vital NETosis occurs quickly after neutrophils are stimulated, and does not require the generation of ROS as well as the activation of the MERK/ERK pathway (Yipp et al., 2012). Currently, how neutrophils commit to one form of NETosis over the other is still unknown, and neutrophil heterogeneity determined by different stimuli is suggested to play a critical role. Since ROS production is essential for suicidal NETosis, it is speculated that neutrophils with an increased level of ROS may produce excessive NETs. Evidence partly supported this notion in which aged CXCR4+ neutrophils produced both increased levels of ROS and NETs (Zhang et al., 2015). In acute inflammation conditions, ICAM-1+ neutrophils and low-density neutrophils which include both mature and immature neutrophils were shown to produce increased amount of NETs (Folco et al., 2018; Kanamaru et al., 2018), although detailed forms of NETs were unknown.

### Beneficial Role of NETs in Sepsis

The earliest evidence of the antimicrobial activities of NETs came from the study which showed that microorganisms were physically attached onto the structural elements of NETs (Buchanan et al., 2006), indicating that NETs exert antimicrobial function by physically trapping microorganisms. Further studies demonstrated that after trapping microorganisms, NETs can directly kill bacteria when phagocytosis of neutrophils was inhibited (Li et al., 2010). Pharmacological inhibition of NET formation by DNase also led to increased burden in the blood, with decreased survival in a mouse CLP model (Czaikoski et al., 2016). Taken together, these results suggested a beneficial role of NETs in the control of invading microorganisms. However, due to their detrimental effects which will be discussed later, the levels of NETosis at different stages of sepsis may impact the outcomes. In supporting this idea, studies showed that administration of DNase at an early time after induction of sepsis by CLP increased pro-inflammatory cytokines and worsened renal and pulmonary injury (Mai et al., 2015). When given at a later stage after CLP, DNase administration reduced organ damage and bacterial dissemination, and also improved survival in the CLP model (Mai et al., 2015). Whether similar results can also be found in human sepsis process are still unknown and need to be verified in the future.

### Detrimental Role of NETs in Sepsis

As mentioned above, NETs protect the host through their antimicrobial activities, while excessive NETosis during sepsis has also been shown to be detrimental to the host through inducing intravascular thrombosis, disseminated intravascular coagulation (DIC), and multiple organ dysfunction. Histones are the most abundant proteins in NETs, and NETs were shown to adhere and activate vascular endothelium during sepsis, which results in endothelium damage in a histone-dependent manner (Clark et al., 2007). Histones can also act as stimulators to TLR signaling pathways which drive the production of pro-inflammatory cytokines (Xu et al., 2009; Allam et al., 2012). Recently, the triggering receptor expressed on myeloid cell-1 (TREM-1) has been identified to potentiate NETosis and impair vascular activity (Murao et al., 2020; Boufenzer et al., 2021). Other components of NETs such as DNA and granule proteins have also been discovered to play a procoagulant role in sepsis (Kannemeier et al., 2007; Massberg et al., 2010; Iba et al., 2014). Furthermore, NETs can intrinsically regulate the coagulation pathway through the activation of factor XII and promote the formation of fibrin (von Bruhl et al., 2012). Despite their detrimental role in the development of coagulation during sepsis, histones from NETs can compromise cell membrane integrity, leading to tissue damage (Abrams et al., 2013). Other NET proteins can also target extracellular matrix proteins, thus disrupting cell junctions (Papayannopoulos, 2018).

The formation of intravascular thrombosis and DIC can lead to microvascular occlusion and tissue hypoxia, which further promote multiple organ failure. Acute respiratory distress syndrome (ARDS) is a common pathophysiological process occurring during sepsis, which is characterized by disruption of the alveolar–capillary barrier. In septic patients, NETs were identified in bronchoalveolar lavage fluid, indicating neutrophils are capable of undergoing NETosis even after transmigration (Yildiz et al., 2015). Serine protease released *via* NETosis such as proteinase-3, cathepsin G, and neutrophil elastase can degrade D and A surfactants, both of which are critical in the resolution of inflammation (Rubio et al., 2004; Cooley et al., 2008). Neutrophil elastase can also increase alveolar epithelial permeability by altering the actin cytoskeleton of epithelial cells (Peterson et al., 1995). DNA released during NETosis such as histones also has a detrimental effect through promoting the destruction of the alveolar epithelium (Bosmann et al., 2013), thus leading to increased severity of ARDS.

### Potential Therapeutic Strategy Targeting Neutrophils for Sepsis Treatment

**FIGURE 1:**
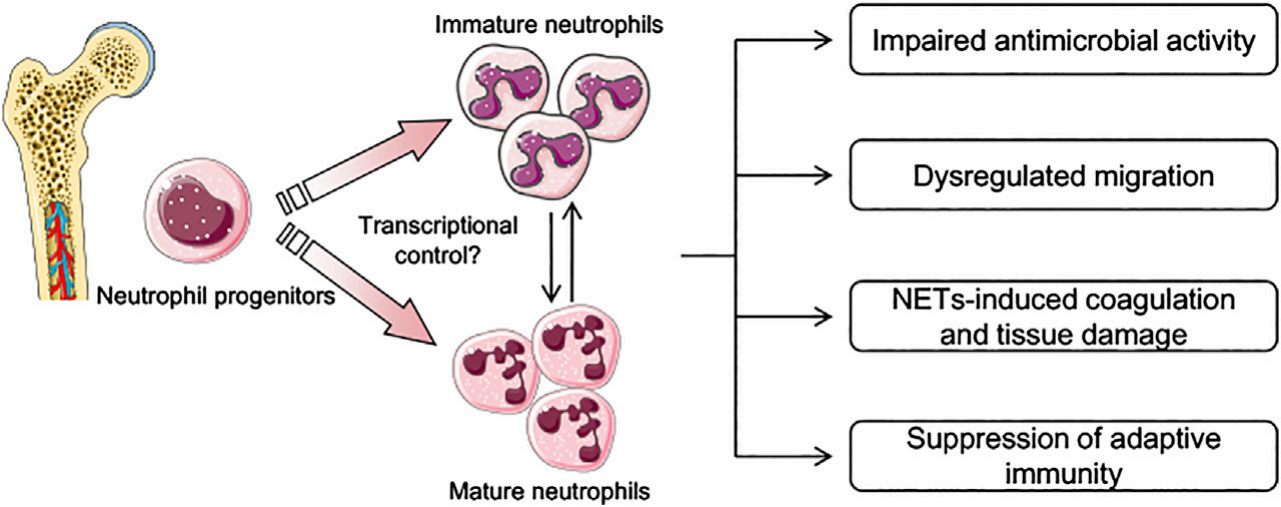
Schematic depicting current knowledge in the pathophysiological features of neutrophils during sepsis. During sepsis, both mature and immature neutrophils are generated in the bone marrow and are mobilized into peripheral blood. Transcriptional factors and environmental stimulus that determine the phenotypes of neutrophils presented in sepsis are largely unknown. Several pathophysiological features related to neutrophil function have been described, including dysregulated migration capacity, impaired antimicrobial activity, and suppression of adaptive immunity through the expression of inhibitory immune checkpoints. Neutrophil extracellular traps (NETs) in sepsis are also not controlled properly, and play an important role in tissue damage and coagulation disturbance.

Currently, treatment of sepsis consists of supportive care and antibiotics, and neither has targeted on the host response which is the main cause of death in sepsis. In addition, how to strengthen immune capacity, which can benefit the control of primary infection and potentially prevent the occurrence of secondary infection, remains to be addressed. Although neutrophils display dysregulated function during sepsis as discussed above, they also have an increased life span which enables them to be a potential therapeutic target (Shen et al., 2017a). Based on the changes identified for neutrophils during the progress of sepsis, how to reverse the inappropriate migration, enhance the antimicrobial capacity, and a better control of NETs may be several potential targeting directions (Figure 1).

Due to their complex function in sepsis, is neutrophil depletion a potential strategy for sepsis treatment? In the severe sepsis animal model established by the CLP procedure, although all the mice succumbed within 72 h, mice with neutrophil depletion displayed a slightly earlier death (Shen et al., 2017b), suggesting an essential role of neutrophils in the control of bacterial infection. Similar results were also discovered in an early study which showed that depletion of neutrophils worsened the outcome in the *Listeria monocytogenes* infection-induced sepsis model (Navarini et al., 2009). Despite their antimicrobial activity, neutrophils also play a detrimental role in remote organ/tissue injury as evidenced by the study, which showed that depletion of neutrophils significantly reduced lung and liver injury despite elevated serum endotoxin levels (Wickel et al., 1997). Therefore, it seems to us that the depletion of neutrophils will only be effective once the primary infection is under control and remote tissue/organ injury has become a central problem that could impact patients’ outcome. In addition, with accumulating results on the identification of neutrophil heterogeneity, targeting a more specific subset of neutrophils that mainly mediate the tissue injury may be a new therapeutic direction. In supporting this idea, targeting an activated phenotype characterized by the expression of CD64 has been suggested to benefit patients’ outcome in septic shock (Lewis et al., 2013).

Granulocyte–macrophage colony-stimulating factor (GM-CSF) is a growth factor which can drive the proliferation and maturation of neutrophils, monocytes, and dendritic cells. Low dose of GM-CSF administration improved oxygenation index with a reduced level of neutrophils and macrophages in the alveoli of patients with sepsis and respiratory dysfunction (Presneill et al., 2002). In patients with nontraumatic abdominal infection, use of GM-CSF also reduced the incidence of infection-related complications (Orozco et al., 2006). Detailed studies further showed that GM-CSF could restore and enhance immunity through manipulating the expression of HLA-DR on monocytes and releasing TNF production from leukocytes (Meisel et al., 2009; Hall et al., 2011). One clinical trial with a large sample size which evaluates the efficacy of GM-CSF for the prevention of secondary infection is now ongoing, which may provide more clinical evidence (NCT02361528). Molecules that target neutrophil maturation and function are also another research field for the development of sepsis treatment. Our group found that wild-type p53-induced phosphatase 1 (Wip1) plays a critical role in neutrophil maturation, and genetic or pharmacological inhibition of Wip1 could increase the infiltration of neutrophils into the primary infection sites and enhance their antimicrobial function (Liu et al., 2013; Shen et al., 2017b; Shen et al., 2017c).

Despite targeting on innate immunity, targeting on adaptive immunity, especially for T-cell immunity, is also another emerging area for the discovery of sepsis treatment. Since neutrophils have been shown to regulate T-cell immune response through the expression of PD-L1 during the early phase of sepsis, it may act a “bridge” between innate and adaptive immunity. Therefore, targeting neutrophils may also lead to beneficial effects on the control of adaptive immunity. Consistent with this, antibodies against inhibitory immune checkpoints such as PD-L1 to restore T-cell function have been evaluated, which showed promising results (Zhang et al., 2010). Furthermore, therapies targeting NET formation and NET components have also been tried, which provide promising prospects. Although early administration of DNase worsened the outcomes in the CLP model, combined treatment of antibiotics and DNase resulted in improved survival, reduced bacteremia, and organ dysfunction (Czaikoski et al., 2016). This indicates combined therapies which include conventional treatment, and NET-targeted drugs can potentially optimize treatment efficacy and outcome in septic patients. Genetic or pharmacological inhibition of other NET formation factors such as PAD4 has been shown to improve survival (Martinod et al., 2015; Biron et al., 2018). However, since NET formation is also important for the control of pathogens, how to maintain an appropriate amount of NETs to meet the demand for bacterial control and also to prevent tissue injury becomes an emerging area for the discovery of targeted therapy. Blockade of NET components such as histones has been shown to provide beneficial effects on survival in the animal model (Semeraro et al., 2011; Li et al., 2014), while large-scale randomized clinical trials evaluating the efficacy for human septic patients failed to demonstrate any clinical benefits (Marti-Carvajal et al., 2012). Recently, other inhibitors targeting extracellular histones have been tried, with promising results identified (Meara et al., 2020).

Because platelet–neutrophil interaction is crucial for NETosis to occur, antiplatelet therapy may also be an interesting field to discover. The activation of platelet requires eicosanoids such as thromboxane A2. Blockade of thromboxane A2 generation with the use of acetylsalicylic acid or aspirin has been shown to decrease intravascular NETosis and tissue injury (Caudrillier et al., 2012). Other inhibitors targeting the platelet–neutrophil interaction such as the platelet ADP receptor P2Y12 also attenuated NETosis (Liverani et al., 2016; Mansour et al., 2020). In addition, in several observational studies, administration of antiplatelet therapy has been shown to be associated with improved outcome in patients with sepsis (Eisen et al., 2012; Chen et al., 2015). Recently, a novel treatment which stabilizes NETs to enhance their capacity of capturing bacteria and prevent the release of antibacterial compounds causing tissue damage has been discovered (Gollomp et al., 2020). Researchers developed an antibody that binds to complexes of NETs and platelet factor 4 (PF4), a protein released by activated platelets, which causes the NETs to resist degradation and improves their ability to capture bacteria. When administered combined with antibiotics, this treatment significantly reduced the severity of illness, decreased the levels of bacteria circulating in the blood, and improved survival in the animal sepsis model (Gollomp et al., 2020). A large-scale clinical trial which evaluates the efficacy of antiplatelet therapy in sepsis is currently underway and may provide interesting results in the future (Eisen et al., 2017; Table 1).

**TABLE 1:** Summary of therapeutic strategy targeting neutrophils.

Target	Strategies	Outcomes	References
Neutrophil	Anti–Gr-1	Reduces remote lung and liver injury in a mice CLP model	Wickel et al. (1997)
	Leucofiltration	Improves organ function in human patients with severe sepsis	Lewis et al. (2013)
	GM-CSF	Drives the maturation and proliferation of neutrophils; Phase III study ongoing	NCT02361528; Meisel et al. (2009), Hall et al. (2011)
	G-CSF	No improvement in clinical trial study	Root et al. (2003)
	Inhibition of Wip1	Drives the maturation of neutrophils and enhances their antimicrobial function	Shen et al. (2017b)
	Anti–PD-L1	Restores T-cell function in the mice model	Zhang et al. (2010)
NETs	DNase I	Effective when combined with antibiotics to improve the outcome	Czaikoski et al. (2016)
	Cl-amidine	Prevents NETs formation and improves survival in a mice CLP model	Biron et al. (2017)
	Anti-citrullinated histone 3	Reduces NETs and improves survival in a mice CLP model	Li et al. (2014)
	Anti–TREM-1	Prevents NETosis and associated endothelial dysfunction in a mice LPS model	Murao et al. (2020), Boufenzer et al. (2021)
Histone	Anti-histone	Improves the outcome in the LPS, TNF, and CLP mice models	Meara et al. (2020)
	Activated protein C	Failed in the clinic	Marti-Carvajal et al. (2012)
Platelet	Acetylsalicylic acid	Decreases intravascular NETosis and tissue injury	Caudrillier et al. (2012)
	Aspirin	Ongoing clinical trial	Eisen et al. (2017)
	Anti-P2Y12	Prevents NETosis and improves the survival in mice CLP model	Liverani et al. (2016), Mansour et al. (2020)
	Antiplatelet factor 4	Stabilizes NETs and prevents the release of antibacterial compounds in mice model	Gollomp et al. (2020)

### Future Perspectives

Sepsis is defined as a dysregulated host response to infection with no specific therapies available currently. As an important component of innate immunity, neutrophils act as sentinels to eliminate invading pathogens and maintain immune homeostasis. With an increased understanding of neutrophil immunity, their role and function during sepsis have been gradually elucidated. Accumulating evidence has also suggested that neutrophils may be a promising therapeutic target for sepsis treatment. However, due to their relatively short half-life and phenotype diversity, intrinsic transcriptional control of neutrophils in severe inflammation such as sepsis is still under investigation (Figure 1). In addition, how environmental stimulus educates and the metabolism control of neutrophils represents another emerging field remain to be discovered. As sepsis is often accompanied with a complex pathophysiological process which involves multiple systems other than immune response, it is also notable that simply “one target” or “one-time-fits-all” approach will unlikely be successful. To achieve dynamic personalized therapy, an easy and effective method to analyze neutrophil function at different phases of sepsis is also important. Interestingly, diagnosis of sepsis based on a single drop of blood assay has been developed, which provides promising results on addressing above issues (Ellett et al., 2018).

### What Is New?

• Despite their antimicrobial function, neutrophils also exert immunoregulatory functions and display phenotypic and functional plasticity.
• An immature form of neutrophils is generated during sepsis, with decreased phagocytic capacity and antimicrobial function. Mechanisms driving the generation of immature neutrophils are unknown, and detailed phenotypes of these immature neutrophils also need to be further explored.
• Neutrophils with effective bacterial phagocytosis can retro-transmigrate from the tissue into the bloodstream. However, these neutrophils are also associated with secondary organ injury at the same time.
• Combined treatment of antibiotics and DNase to prevent excessive NETosis has been shown to improve the outcome of sepsis, suggesting a combination of conventional therapy and treatment targeting on the detrimental effects of neutrophils can potentially optimize treatment efficacy and outcome in septic patients.
• Targeting the platelet–neutrophil interaction may be a new promising therapeutic strategy for sepsis treatment, which has been shown to improve the outcome both in animal and human sepses.
